# The blowflies of the Madeira Archipelago: species diversity, distribution and identification (Diptera, Calliphoridae *s. l.*)

**DOI:** 10.3897/zookeys.634.9262

**Published:** 2016-11-21

**Authors:** Catarina Prado e Castro, Krzysztof Szpila, Anabel Martínez-Sánchez, Rego, Isamberto Silva, Artur R.M. Serrano, Mário Boieiro

**Affiliations:** 1 Centre for Functional Ecology, Department of Life Sciences, University of Coimbra, PT-3000-456 Coimbra, Portugal; 2 Chair of Ecology and Biogeography, Faculty of Biology and Environmental Protection, Nicolaus Copernicus University, Lwowska 1, PL-87-100 Toruń, Poland; 3 Research Institute of Biodiversity CIBIO, Department of Environmental Sciences and Natural Resources, University of Alicante, E-03080 Alicante, Spain; 4 Centre for Ecology, Evolution and Environmental Changes, Azorean Biodiversity Group and Department of Agrarian Sciences, University of Azores, PT-9700-042 Angra do Heroísmo, Azores, Portugal; 5 Madeira Nature Park, PT-9064-512 Funchal, Madeira, Portugal; 6 Centre for Ecology, Evolution and Environmental Changes, Department of Animal Biology, Faculty of Sciences, University of Lisbon, PT-1749-016 Lisbon, Portugal

**Keywords:** Calliphora loewi, island diversity, key, larval description, Laurisilva, Macaronesia

## Abstract

Knowledge on the taxonomic diversity and distribution of blowflies from the Madeira Archipelago is updated. New and interesting findings are reported for poorly studied islands and islets of this archipelago, together with a brief analysis of the diversity of Macaronesian Calliphoridae *s. l.* Seven blowfly species were collected during this study, including the first records of *Calliphora vicina* Robineau-Desvoidy, 1830, *Chrysomya albiceps* (Wiedemann, 1819), *Lucilia sericata* (Meigen, 1826), *Pollenia rudis* (Fabricius, 1794) and *Stomorhina lunata* (Fabricius, 1805) from Porto Santo, and of *Calliphora vicina*, *Lucilia sericata* and *Stomorhina lunata* from Desertas Islands. The presence of *Calliphora loewi* Enderlein, 1903 in Madeira Laurisilva forest is discussed and its first instar larva is redescribed, revealing important differences in relation to its original description. An identification key to the adult Madeiran blowflies is provided for the first time.

## Introduction

Blowflies in the broad sense (Diptera: Calliphoridae, Mesembrinellidae, Rhiniidae) (Kutty et al. 2010, Marinho et al. 2016) are conspicuous flies that can be found in both synanthropic and natural ecosystems. Some species exhibit beautiful metallic colours, ranging from green to violet, while others have a more cryptic colouration. Nevertheless, due to their moderate size and frequent association with domestic and human-disturbed environments, blowflies are usually familiar to people.

There are 115 blowfly species in Europe (Rognes and Baz 2007, Rognes 2013), 12 of these being Rhiniidae, once a subfamily of Calliphoridae *s. l.* but recently raised to the rank of family (Kutty et al. 2010). Recent molecular studies also point to a similar status for the Polleniinae, but this taxon is still treated as a subfamily of Calliphoridae *s. str.* (e.g., Kutty et al. 2010, Singh and Wells 2013, Marinho et al. 2016, Zhang et al. 2016). Adult blowflies have a wide variety of life habits: some species feed on pollen and nectar, playing a role in pollination (Rognes 1998, Pérez-Bañon et al. 2007), while most are usually associated with faeces, animal corpses and other decaying organic matter (Norris 1965, Heath 1982, Rognes 1998). A considerable number of species are parasites or predators of earthworms (*Bellardia* Robineau-Desvoidy, 1863, *Onesia* Robineau-Desvoidy, 1830 and *Pollenia* Robineau-Desvoidy, 1830 species), snails (*Angioneura* Brauer & Bergenstamm, 1893, *Eurychaeta* Brauer & Bergenstamm, 1891 and *Melinda* Robineau-Desvoidy, 1830 species), or parasites of bird nestlings (*Protocalliphora* Hough, 1899 and *Trypocalliphora* Peus, 1960 species) (Rognes 1998). Many blowfly species play a key ecological role as natural agents of decomposition (Erzinçlioglu 1985) and are also important in human and veterinary medicine as transmitters of pathogens and as agents of myiasis (Zumpt 1965, Greenberg 1971, Erzinçlioglu 1985, Rognes 1998). In the Rhiniidae, however, the immature morphology and life cycles are unknown for almost all species, though a predatory/parasitic behaviour in nests of social insects and on orthopteran egg-masses has been either suggested or confirmed (Peris 1952, Rognes 1998, Bharti and Bharti 2016).

The catalogue of Iberian Diptera (Carles-Tolrá 2002), which also includes information on the Canarian, Azorean and Madeiran islands, is a landmark in the study of these insects by providing valuable information on the taxonomic diversity of Diptera at both the national and regional level. Furthermore, the comparison of checklists and associated scientific literature presented in the catalogue clearly highlights disparities in knowledge on many families between the different regions. For example, the poor knowledge of the Portuguese fauna, with only 19 blowfly species recorded (13 Calliphoridae and 6 Rhiniidae) contrasts with the amount of information available for Spain, where 48 blowfly species (41 Calliphoridae and 7 Rhiniidae) are known to occur (Martínez-Sánchez et al. 2002). New findings of blowflies have been reported for continental Portugal (Prado e Castro and García 2009, Prado e Castro et al. 2010) and Spain (Carles-Tolrá 2007) in the last decade, but no significant changes were recorded for the fauna of the Madeira Archipelago. In Portugal, including the Azores and Madeira archipelagos, there is still scarce information on the taxonomic diversity of blowflies due to a lack of national experts and severe limitations in funding for biodiversity inventorying and monitoring programmes targeting invertebrates.

The checklist of Madeiran terrestrial biodiversity (Borges et al. 2008) reports nine species, and recently, *Calliphora loewi* was found for the first time in the archipelago (Prado e Castro et al. 2016). Earlier studies on blowflies from the Madeira Archipelago are scarce and all species records are restricted to the main island (Báez and Santos-Pinto 1975, Rognes 1987, Báez 1990). Herein, an updated checklist is provided of the Calliphoridae and Rhiniidae of the Madeira Archipelago, including new data on the distribution and abundance of seven species. The presence of *Calliphora loewi* Enderlein in Madeira Laurisilva is discussed and its first instar larva is redescribed, showing significant differences in relation to the original description. Finally, a key is provided to enable the identification of adults of all species so far reported from the archipelago.

## Material and methods

### Study area

The Madeira Archipelago is located in the North Atlantic, nearly 600 km from the African coast (Morocco), between latitudes 32°24' and 33°07'N and longitudes 16°16' and 17°16'W. The archipelago consists of three groups of volcanic islands and islets, namely Madeira, Porto Santo and Desertas. Madeira is the largest (~ 740 km^2^) and highest (1862 m) island and also presents the highest diversity of habitat types, including the largest surviving area of Laurisilva forest in Macaronesia. Laurisilva is a relict laurel forest native to the Macaronesian archipelagos of Azores, Canaries and Madeira, which during the Tertiary covered a considerable area of the western Mediterranean Basin (Aguiar et al. 2004). Madeira Island has a considerable number of laurel forest fragments in pristine condition (Neves et al. 1996), which led Madeiran Laurisilva to be classified as a UNESCO World Natural Heritage site (IUCN 1999). Furthermore, mostly due to the unique biodiversity associated with Laurisilva, the Madeira Archipelago was included in one of the most important global biodiversity hotspots, the Mediterranean hotspot (Médail and Quézel 1999, Myers et al. 2000). Porto Santo and Desertas have drier environmental conditions than Madeira. On these islands, the dominant plant cover is of herbaceous communities with some thermophilous shrub species, but there are also a considerable number of areas lacking vegetation and affected by soil erosion.

Protected areas cover a large fraction of the archipelago, aiming to maintain and protect its native biodiversity from a number of human-related threats (Martin et al. 2008, Silva et al. 2008; see also http://www.pnm.pt/). Nevertheless, despite the recognized vulnerability of several endemic terrestrial arthropod species (Martin et al. 2008, Crespo et al. 2014), very few conservation actions specifically targeting this animal group have been carried out.

### Sampling and laboratory work

A sampling programme encompassing different habitat types in all islands of Madeira Archipelago was carried out during the springs and summers of 2011 and 2012 (Table 1). At each site, a combination of sampling techniques (direct sampling, pitfall trapping, vegetation sweeping) were applied to provide an inventory of species of several groups of terrestrial arthropods. Detailed information on the sampling methodology was provided by Serrano et al. (2014). The samples were sorted in the Entomology Laboratory of the Faculty of Sciences, University of Lisbon, Portugal, where adult blowfly specimens were identified to species level using Olympus SZX7 stereomicroscopes and several taxonomic resources (González-Mora and Peris 1988, González-Mora 1989, Peris and González-Mora 1991, Szpila 2012). *Pollenia rudis* specimens were identified, using specific literature (Rognes 1987, 1992), with a Leica M80 at the laboratory of the Department of Environmental Sciences and Natural Resources, University of Alicante, Spain. All identified adult specimens are deposited in the entomological collection of the Department of Animal Biology, University of Lisbon, Portugal.

**Table 1.** Information on sampling dates and localities (site name, habitat type, and geographic coordinates) in the Madeira Archipelago.

Island group	Site name	Habitat type	Latitude (N)	Longitude (W)	Dates
**Madeira**	Abobreiras	Heathland	32°43'13"	16°51'37"	30/V-13/VI/2011
	Achadas da Cruz	*Eucalyptus* plantation	32°50'42"	17°12'25"	26/V-9/VI/2011
	Bica da Cana	*Pinus* plantation	32°44'47"	17°03'25"	26/VI-9/VII/20110
	Calheta 1	Heathland	32°45'28"	17°08'48"	27/V-10/VI/20110
	Calheta 2	*Eucalyptus* plantation	32°45'06"	17°09'14"	27/V-10/VI/20110
	Dunas da Piedade	Dune system	32°44'49"	16°39'27"	2-16/V/20110
	Funduras	Laurisilva	32°44'58"	16°47'30"	31/V-14/VI/20110
	Galhano 1	Laurisilva	32°48'07"	17°09'57"	4-18/VII/20120
	Galhano 3	Laurisilva	32°47'48"	17°10'30"	5-19/VII/20120
	Ilhéu do Farol	Coastal vegetation	32°43'43"	16°39'27"	18/V-1/VI/20110
	Loreto	*Pinus* plantation	32°46'41"	17°12'36"	24/VI-9/VII/20110
	Miradouro das Voltas 1	Laurisilva	32°48'28"	16°57'00"	2-16/VI/20110
	Miradouro das Voltas 2	Laurisilva	32°48'15"	16°56'48"	2-16/VI/20110
	Miradouro das Voltas Ps	*Pseudotsuga* plantation	32°48'43"	16°57'04"	2-16/VI/20110
	Miradouro das Voltas Seq	*Sequoia* plantation	32°48'24"	16°56'47"	2-16/VI/20110
	Montado dos Pessegueiros 2	Laurisilva	32°47'40"	17°05'12"	3-17/VII/20120
	Montado dos Pessegueiros 3	Laurisilva	32°47'44"	17°05'07"	3-17/VII/20120
	Pico das Pedras L	Laurisilva	32°46'08"	16°54'42"	31/V-14/VI/20110
	Pico das Pedras Ps	*Pseudotsuga* plantation	32°46'33"	16°53'48"	31/V-14/VI/20110
	Ponta de S. Lourenço E	Coastal vegetation	32°44'56"	16°41'30"	3-17/V/20110
	Parque eólico	Semi-natural meadow	32°44'45"	16°43'29"	2-16/V/20110
	Ponta de S. Lourenço W	Coastal vegetation	32°44'50"	16°41'55"	3-17/V/20110
	Portela	*Eucalyptus* plantation	32°44'45"	16°49'23"	3-17/VI/20110
	Porto Moniz	*Eucalyptus* plantation	32°50'46"	17°10'37"	24/V-7/VI/20110
	Prazeres	*Pinus* plantation	32°45'58"	17°11'33"	24/VI-9/VII/20110
	Ribeira da Cruz	Laurisilva	32°49'34"	17°12'35"	26/V-9/VI/20110
	Santana	*Pinus* plantation	32°48'09"	16°51'57"	25/VI-10/VII/20110
**Desertas**	Bugio N	Coastal vegetation	32°24'52"	16°28'40"	27/IV-19/V/2011
	Bugio S	Coastal vegetation	32°24'38"	16°28'23"	27/IV-19/V/2011
	Castanheira N	Coastal vegetation	32°33'52"	16°32'12"	26/IV-10/V/20110
	Castanheira S	Coastal vegetation	32°33'11"	16°31'47"	26/IV-10/V/20110
	Eira	Coastal vegetation	32°30'50"	16°30'10"	27/IV-11/V/20110
	Doca	Coastal vegetation	32°31'03"	16°30'41"	26/IV-10/V/20110
	Ilhéu Chão N	Coastal vegetation	32°35'10"	16°32'43"	28/IV-18/V/20110
	Ilhéu Chão S	Coastal vegetation	32°34'52"	16°32'25"	28/IV-18/V/20110
**Porto Santo**	Fonte da Areia	Dune system	33°04'54"	16°21'18"	21/VI-5/VII/20110
	Ilhéu da Cal N	Coastal vegetation	33°0'41"	16°23'07"	22/VI-6/VII/20110
	Ilhéu da Cal S	Coastal vegetation	33°00'7"	16°23'01"	22/VI-6/VII/20110
	Ilhéu do Farol N	Coastal vegetation	33°03'19"	16°17'04"	20/VI-4/VII/20110
	Ilhéu do Farol S	Coastal vegetation	33°03'13"	16°16'43"	20/VI-4/VII/20110
	Ilhéu do Ferro	Coastal vegetation	33°02'16"	16°24'28"	21/VI-5/VII/20110
	Pico Ana Ferreira	*Pinus* plantation	33°02'36"	16°22'24"	21/IV-5/V/20110
	Pico Branco Cup	*Cupressus* plantation	33°05'40"	16°17'55"	23/IV-7/V/20110
	Pico Branco mead	Semi-natural meadow	33°05'29"	16°18'23"	23/IV-7/V/20110
	Pico do Castelo	*Pinus* plantation	33°04'51"	16°19'55"	22/IV-6/V/20110
	Pico do Facho Cup	*Cupressus* plantation	33°05'02"	16°19'17"	22/IV-6/V/20110
	Pico do Facho Pin	*Pinus* plantation	33°04'58"	16°19'28"	22/IV-6/V/20110
	Pico Juliana	*Cupressus* plantation	33°05'33"	16°19'20"	23/IV-7/V/20110

The unexpected finding of larviposition by *Calliphora loewi* in Madeira Laurisilva (Prado e Castro et al. 2016) allowed for a detailed study of its first instar larval morphology. A revision of the first instar larval morphology of forensically important European Calliphorinae species was recently published by Szpila et al. (2014); for a few species, including *Calliphora loewi*, the authors were unable to access original material and had to base their conclusions on information from the literature (Erzinçlioglu 1985). The study of the first instar larva of *Calliphora loewi* was carried out in Poland (Chair of Ecology and Biogeography Laboratory, Faculty of Biology and Environmental Protection, Nicolaus Copernicus University, Toruń) after extraction from dissected, gravid females. Abdomens of females were detached from the rest of the body and boiled in a 5% KOH solution for 5 minutes. Each abdomen was dissected by slicing the membrane between the tergites and sternites, enabling intact first instars to be pulled out of the female oviduct. The larvae were slide-mounted in Hoyer’s medium for study under a light microscope. A Nikon 8400 digital camera, mounted on a Nikon Eclipse E200 microscope, was used for documentation of larval morphology. The larval specimens are housed in the entomological collection of the Chair of Ecology and Biogeography, Nicolaus Copernicus University, Toruń, Poland.

The classification and the taxonomic terminology used in the key follow Rognes (1991), while the terminology used in the description of the first instar larva morphology follows Szpila et al. (2014) and Grzywacz et al. (2014).

## Results

### The blowfly species of the Madeira Archipelago

**Figure 1.**
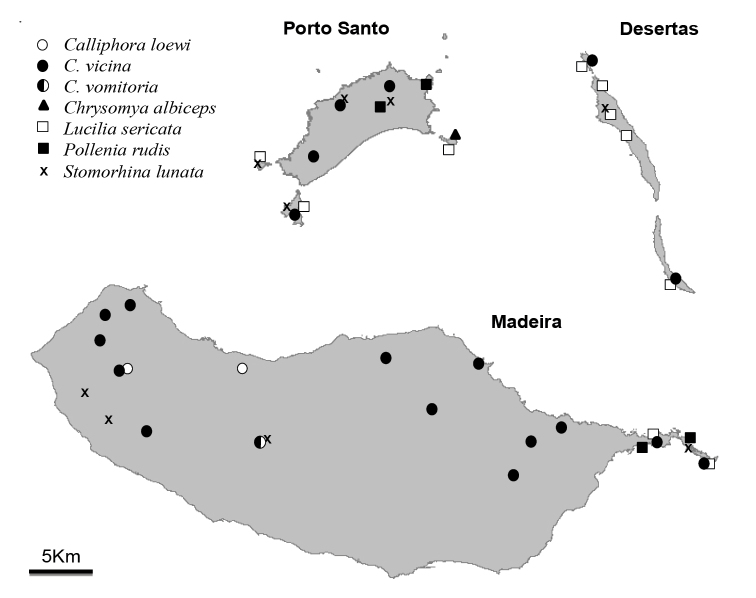
Blowfly records collected during the present study in the islands and islets of the Madeira Archipelago. Species records are plotted on a simplified representation of the archipelago to ease understanding.

Four-hundred and seventy (470) specimens of six Calliphoridae and one Rhiniidae species were collected during this study. The occurrence of blowfly species is reported for the first time from Porto Santo and Desertas. Detailed information associated with the specimens collected in the Madeira Archipelago (sampling date, location, geographic coordinates, habitat-type and number and sex of specimens) is presented under “Material examined” and in Table 1. An updated list of blowfly species from the Madeira Archipelago is presented in Table 2, while the spatial distribution of new species records is shown in Figure 1.

**Table 2.** Updated list of the Calliphoridae and Rhiniidae of the Madeira Archipelago. New records are indicated by a full black circle. M – Madeira Island, PS – Porto Santo Island and surrounding islets, D – Desertas islands (Ilhéu Chão, Deserta Grande and Bugio).

Species	M	PS	D
*Calliphora loewi* Enderlein, 1903	X		
*Calliphora vicina* Robineau-Desvoidy, 1830	X	•	•
*Calliphora vomitoria* (Linnaeus, 1758)	X		
*Chrysomya albiceps* (Wiedemann, 1819)	X	•	
*Chrysomya megacephala* (Fabricius, 1794)	X		
*Lucilia sericata* (Meigen, 1826)	X	•	•
*Pollenia angustigena* Wainwright, 1940	X		
*Pollenia pediculata* Macquart, 1834	X		
*Pollenia rudis* (Fabricius, 1794)	X	•	
*Stomorhina lunata* (Fabricius, 1805)	X	•	•

#### Calliphora loewi

Taxon classificationAnimaliaDipteraCalliphoridae

Enderlein, 1903

Figs 2J , 3A

##### Material examined.

Madeira: Galhano 3 (20 females); Montado dos Pessegueiros 2 (1 female); Montado dos Pessegueiros 3 (3 females).

##### Remarks.

*Calliphora loewi* is a carrion-breeder present in the Holarctic and in a small part of the Oriental Region (Schumann 1986, Verves 2005). It is found in forests of northern and central Europe (Smith 1986, Byrd and Castner 2010), being common in alpine regions. In North America it is found in Alaska, Canada and in the northern continental United States (Rognes 1991, Tantawi et al. in press). Throughout its range, this species is generally not found in urban and disturbed areas (Byrd and Castner 2010). Although widespread, *Calliphora loewi* is rarely recorded, and usually in low abundance, in carcasses of large vertebrates, demonstrating a preference for small animal remains (Szpila et al. 2014). In Madeira, *Calliphora loewi* is restricted to a few native forest areas at high altitude (1000–1300 m) (Fig. 1). This species was recently collected for the first time in Madeira (Prado e Castro et al. 2016).

**Figure 2.**
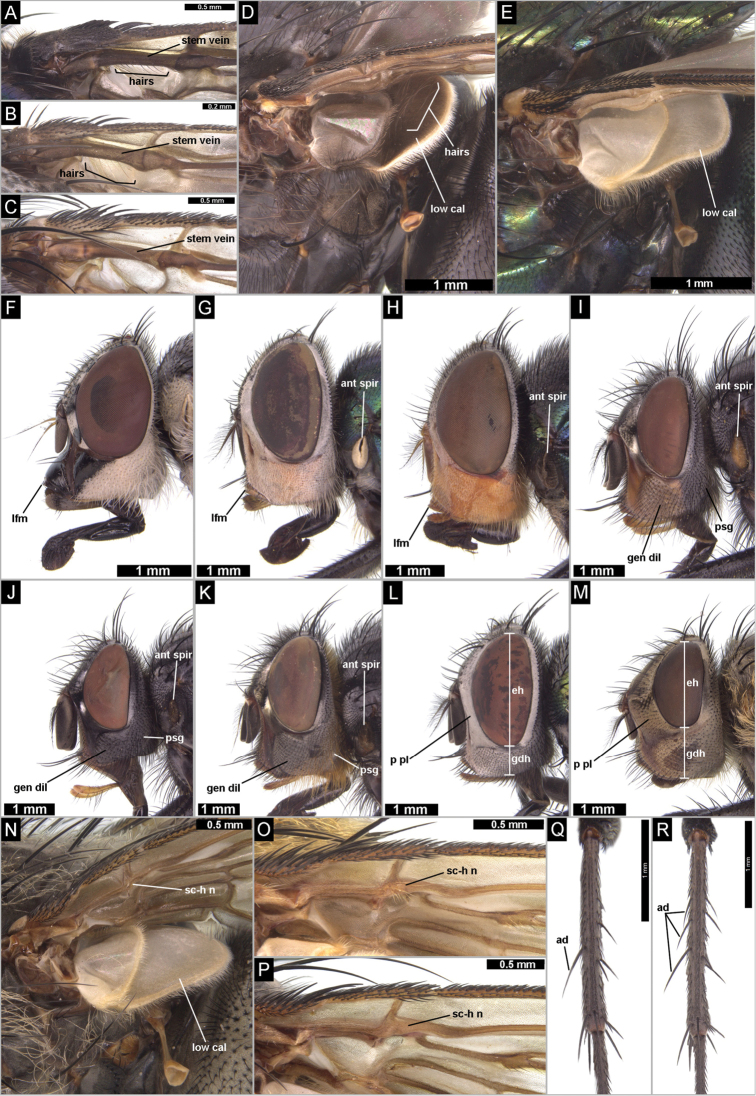
Diagnostically important characters of Madeiran blowflies (Diptera, Calliphoridae *s. l.*): **A** *Chrysomya albiceps*, basal part of wing, dorsal surface, showing haired stem vein **B** *Stomorhina lunata*, basal part of wing, dorsal surface, showing haired stem vein **C** *Calliphora vicina*, basal part of wing, dorsal surface, showing bare stem vein **D** *Calliphora vomitoria*, thorax, upper and lower calypters **E** *Lucilia sericata*, thorax, upper and lower calypters **F** *Stomorhina lunata*, female, head, lateral view **G** *Chrysomya albiceps*, female, head, lateral view **H** *Chrysomya megacephala*, female, head, lateral view **I** *Calliphora vicina*, female, head, lateral view **J** *Calliphora loewi*, female, head, lateral view **K** *Calliphora vomitoria*, female, head, lateral view **L** *Lucilia sericata*, female, head, lateral view **M** *Pollenia rudis*, female, head, lateral view **N** *Pollenia rudis*, thorax, upper and lower calypters **O** *Pollenia pediculata*, basal part of wing, ventral surface, showing haired node of subcostal and humeral veins **P** *Pollenia rudis*, basal part of wing, ventral surface, showing bare node of subcostal and humeral veins **Q** *Pollenia angustigena*, mid tibia **R** *Pollenia rudis*, mid tibia. Abbreviations: **ad**, anterodorsal seta; **ant spir**, anterior spiracle; **eh**, eye height; **gen dil**, genal dilation; **gdh**, genal dilation height; **lfm**, lower facial margin; **low cal**, lower calypter; **p pl**, parafacial plate; **psg**, postgena; **sc-h n**, node subcosta-humeral vein.

**Figure 3.**
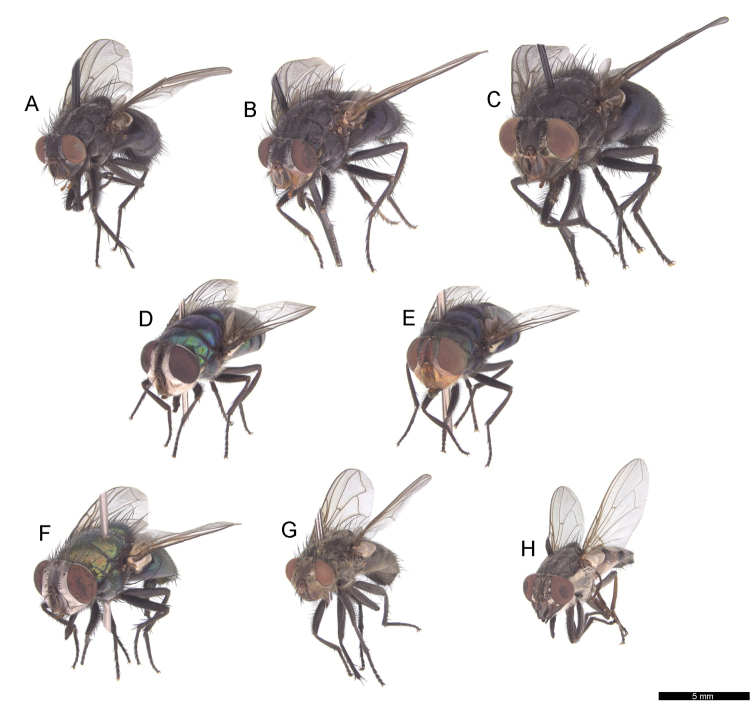
Habitus in antero-lateral view of Madeiran blowflies: **A** *Calliphora loewi* **B** *Calliphora vicina* **C** *Calliphora vomitoria* **D** *Chrysomya albiceps* **E** *Chrysomya megacephala* **F** *Lucilia sericata* **G** *Pollenia rudis* **H** *Stomorhina lunata*.

**Figure 4.**
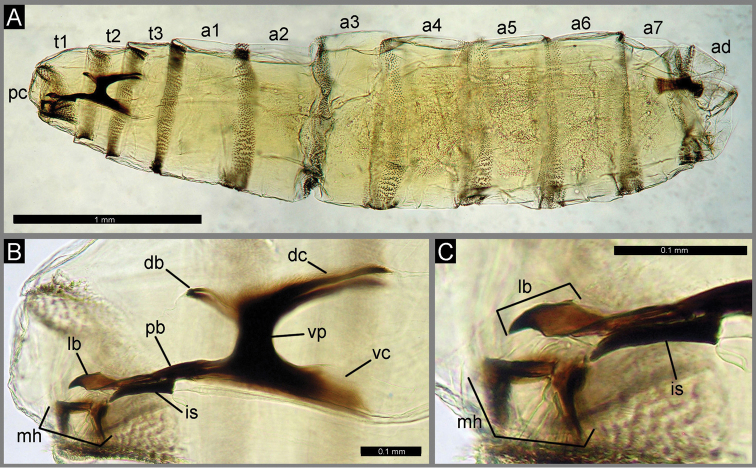
First instar larva of *Calliphora loewi* from Madeira: **A** Habitus in lateral view **B** Cephaloskeleton in lateral view **C** Anterior part of cephaloskeleton in lateral view. Abbreviations: **a1-a7**, abdominal segments; **ad**, anal division; **db**, dorsal bridge; **dc**, dorsal cornua; **is**, intermediate sclerite; **lb**, labrum; **mh**, mouthhook; **pb**, parastomal bar; **pc**, pseudocephalon; **t1-t3**, thoracic segments.

#### Calliphora vicina

Taxon classificationAnimaliaDipteraCalliphoridae

Robineau-Desvoidy, 1830

Figs 2I , 3B

##### Material examined.

Madeira: Abobreiras (1 female); Achadas da Cruz (1 male); Calheta 1 (1 female); Calheta 2 (1 female); Ponta de São Lourenço E (1 male); Ilhéu do Farol (1 male); Funduras (1 female); Galhano 1 (1 female); Miradouro das Voltas 1 (1 female); Miradouro das Voltas 2 (1 female); Miradouro das Voltas Ps (1 female); Miradouro das Voltas Seq (2 females); Montado dos Pessegueiros 2 (1 female, 1 male); Montado dos Pessegueiros 3 (1 female, 2 males); Pico das Pedras L (1 female); Pico das Pedras Ps (2 females); Portela (1 female); Porto Moniz (2 females); Ribeira da Cruz (1 female); Santana (1 female); PORTO SANTO: Ilhéu da Cal S (1 female, 1 male); Fonte da Areia (1 female, 1 male); Pico Ana Ferreira (7 females, 1 male); Pico do Facho Cup (1 female); Pico Juliana (1 female); DESERTAS: Bugio N (12 females, 5 males); Bugio S (7 females, 1 male); Ilhéu Chão N (1 female, 1 male).

##### Remarks.

*Calliphora vicina* is a cosmopolitan species, widely distributed all over the world and closely connected with human activity (Zumpt 1965, Greenberg 1971, González-Mora 1989, Martínez-Sánchez et al. 2002), being commonly found in urban areas (Erzinçlioglu 1985, Rognes 1998, Szpila et al. 2014). The adults are attracted to faeces, meat and fruits, while the larvae are mainly necrophagous, usually developing in carrion (Zumpt 1965, Greenberg 1971). This species is widespread in a variety of habitat types in the Madeira Archipelago (Table 1).

#### Calliphora vomitoria

Taxon classificationAnimaliaDipteraCalliphoridae

(Linnaeus, 1758)

Figs 2K , 3C

##### Material examined.

Madeira: Bica da Cana (1 female).

##### Remarks.

This common carrion-breeder is distributed throughout the Holarctic Region and is also present in the Oriental and Australasian regions (Erzinçlioglu 1985). It is more rural in its distribution than *Calliphora vicina* (Smith 1986), frequenting natural and cultivated forests and showing a preference for more shaded habitats (Szpila et al. 2014). *Calliphora vomitoria* was found only in Madeira, in a pine plantation with low human disturbance.

#### Chrysomya albiceps

Taxon classificationAnimaliaDipteraCalliphoridae

(Wiedemann, 1819)

Figs 2G , 3D

##### Material examined.

Porto Santo: Ilhéu do Farol S (1 female).

##### Remarks.

*Chrysomya albiceps* can be found from the southern Palaearctic Region (southern Europe, Arabia, India) through to Africa (Zumpt 1965) and the Americas, where since its introduction it has rapidly expanded north (Guimarães et al. 1978, Baumgartner and Greenberg 1984). In Europe it is very abundant in the Iberian Peninsula (Martínez-Sánchez et al. 2002, Prado e Castro et al. 2012) and is expanding towards central Europe, having reached France, Switzerland and Austria (Grassberger et al. 2003), the Ukraine (Verves 2004) and Poland, from where its current northernmost records are known (Szpila et al. 2008, Michalski and Szpila in press). Our record from Ilhéu do Farol is the first for Porto Santo. *Chrysomya albiceps* normally breeds in carrion; newly-hatched first instar larvae feed on exudations of decomposing flesh, but the second and third larval stages are facultative predators, feeding also on the larvae of other blowfly species (Zumpt 1965).

#### Chrysomya megacephala

Taxon classificationAnimaliaDipteraCalliphoridae

(Fabricius, 1794)

Figs 2H , 3E

##### Records.

Madeira: Funchal (Báez 1990).

##### Remarks.

*Chrysomya megacephala* is widely distributed over the Oriental and Australasian regions, also occurring in many neighbouring parts of the Palaearctic Region (Zumpt 1965). It was introduced in South America (Guimarães et al. 1978), probably from southern Africa (Baumgartner and Greenberg 1984), and into the United States (Greenberg 1988). In Europe it is known from Spain (Martínez-Sánchez et al. 2001), Portugal (Prado e Castro and García 2009), Malta (Ebejer 2007) and from the archipelagos of Madeira (Báez 1990) and the Canaries (Báez et al. 1981). *Chrysomya megacephala* is normally a faeces and carrion breeder (Zumpt 1965), considered a dangerous dipteran vector of pathogens (Wells 1991) and a major pest of fish products (Wall et al. 2001).

#### Lucilia sericata

Taxon classificationAnimaliaDipteraCalliphoridae

(Meigen, 1826)

Figs 2L , 3F

##### Material examined.

Madeira: Dunas da Piedade (1 male); Ilhéu do Farol (1 female); Porto Santo: Ilhéu da Cal S (1 male); Ilhéu do Farol N (1 female, 5 males); Ilhéu do Farol S (2 females); Ilhéu do Ferro (1 female); Desertas: Bugio N (130 females, 30 males); Bugio S (116 females, 34 males); Castanheira N (13 females, 5 males); Castanheira S (3 females); Doca (10 females, 5 males); Eira (1 female); Ilhéu Chão N (1 female); Ilhéu Chão S (1 female).

##### Remarks.

A very common fly in temperate areas of the Holarctic Region (Zumpt 1965). It is practically cosmopolitan, widespread throughout the major zoogeographical regions (Smith 1986, Rognes 1991), and is directly connected to human activity (Martínez-Sánchez et al. 2002). *Lucilia sericata* was found in all island groups of the Madeira Archipelago where it was occasionally recorded in high abundance. The adults are attracted mainly to carrion or open wounds (usually with necrotic tissues) (Zumpt 1965).

#### Pollenia angustigena

Taxon classificationAnimaliaDipteraCalliphoridae

Wainwright, 1940

##### Records.

Madeira: Madeira (Rognes 1987).

##### Remarks.

This species is cited from most countries of Europe and from the Nearctic Region (Rognes 1991). In Europe, *Pollenia angustigena* can be found from the southern parts of Norway and Finland to Italy and the Iberian Peninsula (Martínez-Sánchez et al. 2002). In the Iberian Peninsula the species seems to be restricted to high altitudes (from 300 m to above 1200 m) (Martínez-Sánchez et al. 1998). No specimens were caught in this study but the species was previously cited from Madeira (Rognes 1987, Martínez-Sánchez and Rognes 2008). Adults of this fly are active mostly from March to October with peaks in early spring in the northern parts of its range (Rognes 1991). As the rest of *Pollenia* spp., it is a predator on earthworm species (Rognes 1987). The egg and first instar larva of this species were described by Grzywacz et al. (2012) and Szpila (2003), respectively, and their morphology is significantly different compared to that of necrophagous blowflies.

#### Pollenia pediculata

Taxon classificationAnimaliaDipteraCalliphoridae

Macquart, 1834

##### Records.

Madeira: Madeira (Rognes 1987).

##### Remarks.

This species is distributed throughout most zoogeographical regions, being widespread in Europe. *Pollenia pediculata* was first cited from Madeira by Rognes (1987), but no specimens were obtained in the present study. The adults are active mainly in summer (Martínez-Sánchez et al. 2001). The larvae are predators on earthworms, including the lumbricid *Eisenia rosea* (Savigny) (Rognes 1987). The early immature stages were described by Szpila (2003) and Grzywacz et al. (2012).

#### Pollenia rudis

Taxon classificationAnimaliaDipteraCalliphoridae

(Fabricius, 1794)

Figs 2M , 3G

##### Material examined.

Madeira: Parque eólico (2 females); Ponta de São Lourenço W (1 female); Porto Santo: Pico Branco Cup (6 females); Pico Branco mead (4 females); Pico do Castelo (4 females).

##### Remarks.

This is the most common species in the genus, being widespread in the Palaearctic, Nearctic and Oriental regions. In Porto Santo it was found in different habitat types (Table 1). *Pollenia rudis* uses earthworms as larval hosts and is active all year round, particularly during spring (Rognes 1987, Martínez-Sánchez et al. 1998). The immature stages were described by Yahnke and George (1972), Szpila (2003) and Grzywacz et al. (2012).

#### Stomorhina lunata

Taxon classificationAnimaliaDipteraCalliphoridae

(Fabricius, 1805)

Figs 2F , 3H

##### Material examined.

Madeira: Bica da Cana (2 females); Loreto (3 females); Prazeres (1 female); Parque eólico (1 female); Ponta de São Lourenço W (1 female); Porto Santo: Ilhéu da Cal N (4 females); Ilhéu da Cal S (2 females); Ilhéu do Ferro (1 female); Fonte da Areia (7 females); Pico do Facho Pin (1 female); Desertas: Castanheira N (1 female).

##### Remarks.

*Stomorhina lunata* is distributed almost worldwide, being absent only from the Neotropical and Australasian regions (Rognes 2013). It is common in Europe including all of the Mediterranean area, and is widely distributed throughout the Iberian Peninsula (González-Mora and Peris 1988). It is known as a predator on egg-masses of the locust *Schistocerca gregaria* (Forskål, 1775) (Rognes 1998) and a parasite in nests of ants (Bharti and Bharti 2016). Adults are commonly found on flowers (González-Mora and Peris 1988).

### Key to the adult blowflies of the Madeira Archipelago

**Table:** 

1	Stem-vein with a row of weak setulae on dorsal surface (Fig. 2A, B)	**2**
–	Stem-vein bare on dorsal surface (Fig. 2C)	**4**
2	Lower facial margin strongly protruded (Fig. 2F); arista bare on ventral side (Fig. 2F); abdomen with a yellow pattern on black background	***Stomorhina lunata***
–	Lower facial margin not protruded (Fig. 2G, H); arista always with hairs dorsally and ventrally (Fig. 2G, H); abdomen lustrous, bluish or green, with dark transverse strips on posterior margins of segments	**3**
3	Anterior spiracle light-coloured, white-yellowish (Fig. 2G)	***Chrysomya albiceps***
–	Anterior spiracle dark, brownish (Fig. 2H)	***Chrysomya megacephala***
4	Lower calypter black or at least darkly infuscate, with numerous long hairs on dorsal surface (Fig. 2D)	**5**
–	Lower calypter white-yellowish and bare on dorsal surface (Fig. 2E, N)	**7**
5	Facial ridge, lower facial margin and anterior part of genal dilation yellowish-red (Fig. 2I); anterior spiracle (Fig. 2I) and basicosta light-coloured, brownish	***Calliphora vicina***
–	Facial ridge, lower facial margin and anterior part of genal dilation black (Fig. 2J, K); anterior spiracle and basicosta black (Fig. 2J, K)	**6**
6	Hairs on posterior part of genal dilation and postgena black (Fig. 2J)	***Calliphora loewi***
–	Hairs on posterior part of genal dilation and postgena orange (Fig. 2K)	***Calliphora vomitoria***
7	Parafacial plates narrow and bare, with white dusting (Figs 2L, 3F); height of genal dilation distinctly shorter than half of eye height (Fig. 2L); body lustrous green (Fig. 3F)	***Lucilia sericata***
–	Parafacial plates broad and densely haired, with brown-yellowish dusting (Fig. 2M); height of genal dilation distinctly longer than half of eye height (Fig. 2M); body dark, abdomen with grey tessellated pattern	**8**
8	Node at junction of humeral crossvein and subcostal vein with a bundle of several light-coloured hairs (Fig. 2O)	***Pollenia pediculata***
–	Node at junction of humeral crossvein and subcostal vein bare (Fig. 2N, P)	**9**
9	Mid tibia with one anterodorsal seta (Fig. 2Q)	***Pollenia angustigena***
–	Mid tibia with two or three anterodorsal setae (Fig. 2R)	***Pollenia rudis***

### Redescription of the first instar larva of *Calliphora loewi*

#### Calliphora loewi

Taxon classificationAnimaliaDipteraCalliphoridae

Enderlein, 1903

##### Material examined.

Madeira: Galhano 3 (15 first instar larvae). The first instar larvae (Fig. 4A) were obtained from 13 gravid females collected in Galhano (Prado e Castro et al. 2016).

##### Diagnosis.

The first instar larvae of *Calliphora loewi* from Madeira possess the general habitus characteristic of most Calyptratae, being divided into a bilobed pseudocephalon (pc), three thoracic segments (t1–t3), seven abdominal segments (a1–a7), and an anal division (ad) that carries the posterior spiracles.

##### Redescription.

Body length: 1.4–5.1 mm. *Pseudocephalon*. Antennal complex with small antennal dome situated on basal ring, antennal dome slightly longer than height of basal ring; maxillary palpus located on anterior surface of pseudocephalic lobe and readily visible under a light microscope as a flat disc clearly distinguished from the surrounding cuticular surface; oral ridges present from lateral margins of functional mouth opening to ventral and lateral surfaces of pseudocephalon; functional mouth opening with two lateral tufts of numerous cirri. *Cephaloskeleton*. As in other necrophagous blowflies; consisting of unpaired labrum (lb), paired mouthhooks (mh), unpaired and H-shaped intermediate sclerite (is) and basal sclerite with parastomal bars (pb), vertical plates (vp) and ventral and dorsal cornua (vc, dc) (Figs 4B, C); each mouthhook an L-shaped bar, with tip equipped with 6 strong, pointed teeth directed ventrally, teeth arranged in one row with one tooth situated in front of row (well visible in ventral view); basal part of mouthhook also strongly sclerotized and equipped with a lateral arm (Figs 4B, C); labrum with readily differentiated sharp and curved apical part, ventral incision separating apical and basal parts of labrum indistinct (Fig. 4C); epistomal sclerite [“posterior expansion of labrum” in Szpila et al. (2014)] flat in lateral view (Fig. 4C); parastomal bars (pb) long and slender; intermediate sclerite (is) and crossbeam of intermediate sclerite broad; vertical plate (vp) as broad as width of ventral cornua (Fig. 4B); dorsal cornua slightly longer than ventral cornua (Fig. 4B); dorsal bridge present (Fig. 4B). *Thoracic segments* Anterior spinose band on first thoracic segment broad (Fig. 4A), with spines arranged in 5–7 rows dorsally and 12–14 rows ventrally; anterior spinose bands of second and third thoracic segments with homogenous conical, slightly flattened spines, tip of spines slightly curved. *Abdominal segments*. Anterior spinose bands complete on segments a1–a5, narrowly interrupted dorsally on segment a6; segment a7 with anterior spinose band on ventral and ventro-lateral surfaces and with several spines on lateral surface; posterior spinose band on segment a1 reduced to two small groups of spines situated ventro-laterally, on a2 posterior spines only on ventral and ventro-lateral surfaces, segment a3 with narrow posterior spinose band interrupted dorsally, segments a4–a7 with complete posterior spinose band. *Anal division*. Anal pads rounded and slightly protruding, anal tuft with several spines dorsally; circle of hair-like spines around spiracular field complete; anterior spinose band developed only on ventral and ventro-lateral surfaces.

##### Comparison with original description.

The comparison of first instar larval specimens from Madeira with the original description (Erzinçlioğlu 1985) points to several discrepancies. Erzinçlioğlu (1985) described the anterior spinose band on a5 of *Calliphora loewi* as interrupted dorsally. Szpila et al. (2014) used this character for separation of *Calliphora loewi* larvae from those of *Calliphora vicina* and *Cynomya mortuorum* (Linnaeus, 1761), where the anterior spinose band on a5 is complete. However, the first instar larvae of *Calliphora loewi* from Madeira also possess a complete anterior spinose band on a5. This character seems to be variable and cannot be treated as reliable. Serious discrepancies between the material from Madeira and the British larvae studied by Erzinçlioğlu (1985) were also found in the morphology of the labrum in the cephaloskeleton. Larvae from Madeira possess a massive labrum with broad basal part and well differentiated apical part. Additionally, the apical part of the labrum is noticeably curved downward. The cephaloskeleton of *Calliphora loewi* larvae in the schematic illustration provided by Erzinçlioğlu (1985, fig. 28) possesses an elongated labrum (labelled as “median tooth”), without clear differentiation between an apical part and a basal part. Unfortunately, at this stage it is not possible to state that these differences between Madeira specimens and the original description result from interpopulation variation or inaccuracy of observation, as the larval material analyzed for the original description is unavailable (Szpila et al. 2013). Future studies on the variation of *Calliphora loewi* larval morphology across the species distribution range will help to clarify this issue.

## Discussion

The effective conservation of insect diversity is, to some extent, hindered by the lack of knowledge of species taxonomy as well as by poor data on species distribution, abundance and sensitivity to habitat change (e.g., Cardoso et al. 2011, New 2012). Consequently, major efforts have been made during the last decade to overcome these obstacles, leading to the production of updated catalogues of species and the identification of conservation priorities for many countries and regions in the world (e.g., Martín et al. 2008, Verdú and Galante 2009, Van Swaay et al. 2010).

This recent survey of the calliphorid diversity on the islands and islets of the Madeira Archipelago allowed the collection of valuable information concerning species taxonomy, distribution and ecology, including the first report of blowfly species from Porto Santo and Desertas islands (Table 2). The number of Calliphoridae *s. l.* species known from the Madeira Archipelago is presently ten, but there is a considerable difference in calliphorid species richness between islands: Madeira (10 spp.), Porto Santo (5 spp.) and Desertas (3 spp.). The lower number of blowfly species recorded from Porto Santo and Desertas is not only due to geographic and ecological determinants (e.g. lower surface area, lower habitat diversity), but also a result of undersampling. Our blowfly survey was the first carried out in Porto Santo and Desertas. These islands have traditionally been disregarded by taxonomic experts, who have focused their sampling efforts exclusively on the main island when visiting the archipelago. Consequently, the present knowledge of Madeiran biodiversity is biased and efforts should be addressed to develop inventory programmes exploring the taxonomic biodiversity of also Porto Santo and Desertas.

This study presents evidence that the species found in the Madeira Archipelago use different habitats: *Calliphora vicina* was the most widespread blowfly, occurring in a variety of habitat types (natural, semi-natural and forest plantations) and ranging from thermophilous coastal areas to native forest patches in valleys and mountainous areas. Three other species (*Lucilia sericata*, *Pollenia rudis*, *Stomorhina lunata*) were more common in coastal areas and inland forest plantations, while the two remaining *Calliphora* species (*Calliphora loewi* and *Calliphora vomitoria*) were restricted to a few forest locations at higher altitudes. A similar pattern of calliphorid species distribution was found in the Canaries, where *Calliphora vicina* was considered an “extreme habitat generalist”, *Lucilia sericata* seemed to be restricted to open habitats at low altitudes and *Calliphora splendens* Macquart, 1839 and *Calliphora vomitoria* were rare and confined to forest habitats (Hanski 1977).

The finding of *Calliphora loewi* in the Madeira Archipelago was surprising, since in Europe the species is known to occur mainly in forest ecosystems at much higher latitudes (Smith 1986, Byrd and Castner 2010) and is absent from the Azores and the Canary Islands (Arechavaleta et al. 2010, Borges et al. 2010). It is presumed that the Madeiran populations of *Calliphora loewi* are relictual due to their isolation from the nearest mainland populations and considering the historical biogeography of Laurisilva, a laurel forest type once distributed throughout the western Mediterranean and which is now restricted to Macaronesia (Neves et al. 1996). Furthermore, the finding that the Madeiran populations of *Calliphora loewi* are strictly associated with pristine Laurisilva patches at high altitudes also seems to support their relictual origin: the two Laurisilva patches where *Calliphora loewi* was found, Montado dos Pessegueiros and Galhano, are well preserved, have minimal human disturbance, and show similar plant compositions (Neves et al. 1996). Like *Calliphora loewi*, the Canarian endemic *Calliphora splendens* is also restricted to native forest areas at higher altitudes (above 400 m) being absent from disturbed, low altitude areas where introduced calliphorids dominate (Hanski 1977, Báez 1988).

The number of calliphorid species so far recorded for the Madeira Archipelago remains low, but it is similar to numbers reported from the Azores (nine species) and Canary Islands (ten species) (Báez and Oromí 2010, Martínez-Sánchez 2010). The low species richness of these oceanic archipelagos is in part due to the difficulties faced in overcoming the ecological barrier posed by the ocean, even for insects with a good flight capacity such as blowflies. Moreover, according to Hanski (1977), the most likely explanation for the impoverished calliphorid faunas of Madeira and the Canaries is the intense interspecific competition due to low carrion density and diversity in these isolated ecosystems, which severely constrains species richness and abundance of the carrion breeding species.

During the last decades we have witnessed a significant increase in the number of blowfly introductions in oceanic archipelagos worldwide due to human-assisted dispersal (Hanski 1977, Báez et al. 1981, Báez 1990, Peck et al. 1998, Jensen et al. 2013). The dramatic increase in the frequency of transport of human commodities to islands (Pombo et al. 2010) is a serious challenge for local authorities that have to deal with the consequences of undesired introductions of pathogens, agricultural and forest pests, and disease vectors such as calliphorid species of veterinary or medical importance (Heath and Bishop 2006). The checklists of Calliphoridae *s. l.* of the Madeira, Azores and Canaries archipelagos show that, as a result of human activities, introduced species are well represented, with six species being common to the three archipelagos and widespread in each of them (Martínez-Sánchez and Rognes 2008, Báez and Oromí 2010, Martínez-Sánchez 2010). For example, the introduction of *Chrysomya megacephala* in Tenerife was followed by its expansion within this island and to other Canary Islands (Gran Canaria, Fuerteventura and Lanzarote), andit was later found also in Madeira (Báez et al. 1981, Báez 1990). The increase in blowfly species introductions has led to the increased homogenization of the faunas of the Macaronesian islands, but its consequences for native biodiversity remain unstudied (Hanski 1977). Thus, efforts should be made to implement monitoring programmes to evaluate the conservation status of native blowfly species in Macaronesia, particularly the Canarian endemic *Calliphora splendens* and the recently discovered *Calliphora loewi*, and assess their vulnerability to introduced species.

## Supplementary Material

XML Treatment for Calliphora loewi

XML Treatment for Calliphora vicina

XML Treatment for Calliphora vomitoria

XML Treatment for Chrysomya albiceps

XML Treatment for Chrysomya megacephala

XML Treatment for Lucilia sericata

XML Treatment for Pollenia angustigena

XML Treatment for Pollenia pediculata

XML Treatment for Pollenia rudis

XML Treatment for Stomorhina lunata

XML Treatment for Calliphora loewi

## References

[B1] Aguiar C Capelo J Costa JC Fontinha S Espírito-Santo D Jardim R Lousa M Rivas-Martinez S Mesquita S Sequeira M Sousa J (2004) A paisagem vegetal da Ilha da Madeira. Quercetea 6: 3–200.

[B2] Arechavaleta M Rodríguez S Zurita N García A (Eds) (2010) Lista de especies silvestres de Canarias. Hongos, plantas y animales terrestres. 2009 Gobierno de Canarias, 579 pp.

[B3] Báez M (1988) Análisis faunístico de los Dípteros de la laurisilva de Tenerife, Islas Canarias (Insecta, Diptera). Boletín Asociación española de Entomología 12: 181–208.

[B4] Báez M (1990) Immigration of the oriental latrine fly, *Chrysomya megacephala* (Fabricius), into Madeira (Diptera, Calliphoridae). Bocagiana 141: 1–2.

[B5] Báez M Oromí P (2010) Planipennes, Trichoptera, Lepidoptera, Diptera, Hymenoptera. In: Arechavaleta M Rodríguez S Zurita N García (Eds) Lista de especies silvestres de Canarias. Hongos, plantas y animales terrestres. Gobierno de Canarias, 302–366.

[B6] Báez M Ortega G Kurahashi H (1981) Immigration of the Oriental Latrine Fly, *Chrysomya megacephala* (Fabricius) and the Afrotropical Filth Fly *Ch. chloropyga* (Wiedemann), into the Canary Islands (Diptera: Calliphoridae). Kontyu 49: 712–714.

[B7] Báez M Santos-Pinto E (1975) Dipteros de Canarias. I: Calliphoridae. Vieraea 5: 1–22.

[B8] Baumgartner DL Greenberg B (1984) The genus *Chrysomya* (Diptera: Calliphoridae) in the New World. Journal of Medical Entomology 21: 105–113. doi: 10.1093/jmedent/21.1.105

[B9] Bharti M Bharti H (2016) Association and impact of ectoparasitic blowflies (Diptera: Calliphoridae) on Himalayan ants of genus *Myrmica*. Insectes Sociaux 63: 477–480. doi: 10.1007/s00040-016-0480-4

[B10] Borges PAV Abreu C Aguiar AMF Carvalho P Jardim R Melo I Oliveira P Sérgio C Serrano ARM Vieira P (Eds) (2008) A list of the terrestrial fungi, flora and fauna of Madeira and Selvagens archipelagos. Direcção Regional do Ambiente da Madeira and Universidade dos Açores, Funchal and Angra do Heroísmo, 440 pp http://www.azoresbioportal.angra.uac.pt/files/publicacoes_Listagem%20dMadeira%20e%20Selvagens.pdf

[B11] Borges PAV Costa A Cunha R Gabriel R Gonçalves V Martins AF Melo I Parente M Raposeiro P Rodrigues P Santos RS Silva L Vieira P Vieira V (Eds) (2010) A list of the terrestrial and marine biota from the Azores. Princípia, Oeiras, 432 pp.

[B12] Byrd JH Castner JL (2010) Insects of forensic importance. In: Byrd JH Castner JL (Eds) Forensic entomology. The utility of arthropods in legal investigations. CRC Press, Boca Raton, 39–126.

[B13] Cardoso P Erwin TL Borges PAV New TR (2011) The seven impediments in invertebrate conservation and how to overcome them. Biological Conservation 144: 2647–2655. doi: 10.1016/j.biocon.2011.07.024

[B14] Carles-Tolrá M (Ed.) (2002) Catálogo de los Diptera de España, Portugal y Andorra (Insecta). Monografías de la Sociedad Entomológica Aragonesa 8, Zaragoza, 323 pp http://www.sea-entomologia.org/PDF/MSEA08.pdf

[B15] Carles-Tolrá M (2007) Géneros y especies nuevos para España (Diptera: Limoniidae, Asilidae, Heleomyzidae, Psilidae, Calliphoridae). Heteropterus Revista de Entomología 7(1): 107–109.

[B16] Crespo LC Silva I Borges PAV Cardoso P (2014) Assessing the conservation status of the strict endemic Desertas wolf spider, *Hogna ingens* (Araneae, Lycosidae). Journal for Nature Conservation 22: 516–524. doi: 10.1016/j.jnc.2014.08.005

[B17] Ebejer MJ (2007) The occurrence of *Chrysomya megacephala* (Fabricius) (Diptera, Brachycera) in Malta and records of other Calliphoridae from the Maltese Islands. Entomologist’s Monthly Magazine 143: 165–170.

[B18] Erzinçlioğlu YZ (1985) Immature stages of British *Calliphora* and *Cynomya*, with a reevaluation of the taxonomic characters of larval Calliphoridae (Diptera). Journal of Natural History 19: 69–96. doi: 10.1080/00222938500770041

[B19] González Mora D (1989) Los Calliphoridae de España. II: Calliphorini (Diptera). Eos 65: 39–59.

[B20] González-Mora D Peris SV (1988) Los Calliphoridae de España. I: Rhiniinae y Chrysomynae (Diptera). Eos 64: 91–139.

[B21] Grassberger M Friedrich E Reiter C (2003) The blowfly *Chrysomya albiceps* (Wiedemann) (Diptera: Calliphoridae) as a new forensic indicator in Central Europe. International Journal of Legal Medicine 117: 75–81. 12690503 10.1007/s00414-002-0323-x

[B22] Greenberg B (1971) Flies and disease I: Ecology, classification and biotic associations. Princeton University Press, New Jersey, 856 pp.

[B23] Greenberg B (1988) *Chrysomya megacephala* (F.) (Diptera: Calliphoridae) collected in North America and notes on *Chrysomya* species present in the New World. Journal of Medical Entomology 25: 199–200. doi: 10.1093/jmedent/25.3.199 3392717 10.1093/jmedent/25.3.199

[B24] Grzywacz A Góral T Szpila K Hall MJR (2014) Confocal laser scanning microscopy as a valuable tool in Diptera larval morphology studies. Parasitology Research 113: 4297–4302. doi: 10.1007/s00436-014-4125-0 25231077 10.1007/s00436-014-4125-0 PMC4200345

[B25] Grzywacz A Szpila K Pape T (2012) Egg morphology of nine species of *Pollenia* Robineau-Desvoidy, 1830 (Diptera: Calliphoridae). Microscopy Research and Technique 75: 955–967. doi: 10.1002/jemt.22020 22371287 10.1002/jemt.22020

[B26] Guimarães JH Prado AP Linhares AX (1978) Three newly introduced blowfly species in southern Brazil (Diptera: Calliphoridae). Revista Brasileira de Entomologia 22: 53–60.

[B27] Hanski I (1977) Biogeography and ecology of carrion flies in the Canary Islands. Annales Entomologici Fennici 43: 101–107.

[B28] Heath ACG (1982) Beneficial aspects of blowflies (Diptera: Calliphoridae). New Zealand Entomologist 7: 343–348. doi: 10.1080/00779962.1982.9722422

[B29] Heath ACG Bishop DM (2006) Flystrike in New Zealand: an overview based on a 16-year study, following the introduction and dispersal of the Australian sheep blowfly, *Lucilia cuprina* Wiedemann (Diptera: Calliphoridae). Veterinary Parasitology 137: 333–344. doi: 10.1016/j.vetpar.2006.01.006 16464534 10.1016/j.vetpar.2006.01.006

[B30] IUCN (1999) IUCN evaluation of nominations of natural and mixed properties to the World Heritage List. Bureau of the World Heritage Committee, Paris http://whc.unesco.org/archive/1999/whc-99-conf204-inf8e.pdf

[B31] Jensen J-K Hansen JF Nolsø Á (2013) Blowflies (Diptera, Calliphoridae) of the Faroe Islands, species list and collection sites. Norwegian Journal of Entomology 60: 1–7.

[B32] Kutty SN Pape T Wiegmann BM Meier R (2010) Molecular phylogeny of the Calyptratae (Diptera: Cyclorrhapha) with an emphasis on the superfamily Oestroidea and the position of Mystacinobiidae and McAlpine’s fly. Systematic Entomology 35: 614–635. doi: 10.1111/j.1365-3113.2010.00536.x

[B33] Marinho MAT Wolff M Ramos-Pastrana Y Azeredo-Espin AML Amorim DS (2016) The first phylogenetic study of Mesembrinellidae (Diptera: Oestroidea) based on molecular data: clades and congruence with morphological characters. Cladistics. doi: 10.1111/cla.12157 10.1111/cla.12157 34710970

[B34] Martín JL Arechavaleta M Borges PAV Faria B (Eds) (2008) Top 100 – As cem espécies ameaçadas prioritárias em termos de gestão na região europeia biogeográfica da Macaronésia. Consejer’a de Medio Ambiente y Ordenación Territorial, Gobierno de Canarias, 500 pp http://cita.angra.uac.pt/ficheiros/publicacoes/1258124693.pdf

[B35] Martínez-Sánchez A (2010) Diptera (Calliphoridae). In: Borges PAV Costa A Cunha R Gabriel R Gonçalves V Martins AF Melo I Parente M Raposeiro P Rodrigues P Santos RS Silva L Vieira P Vieira V (Eds) A list of the terrestrial and marine biota from the Azores, Principia, Cascais, 233 pp.

[B36] Martínez-Sánchez A Marcos-García MA Rojo S (2001) First collection of *Chrysomya megacephala* (Fabr.) in Europe (Diptera: Calliphoridae). Pan-Pacific Entomologist 77: 240–243.

[B37] Martínez-Sánchez A Rognes K (2008) Calliphoridae. In: Borges PAV, Abreu C, Aguiar AMF, Carvalho P, Jardim R, Melo I, Oliveira P, Sérgio C, Serrano ARM, Vieira P (Eds) A list of the terrestrial fungi, flora and fauna of Madeira and Selvagens archipelagos. Direcção Regional do Ambiente da Madeira and Universidade dos Açores, Funchal and Angra do Heroismo, 329 pp.

[B38] Martínez-Sánchez A Rognes K Báez M (2002) Calliphoridae. In: Carles-Tolrá M (Ed.) Catálogo de los Diptera de España, Portugal y Andorra (Insecta). Monografías de la Sociedad Entomológica Aragonesa 8, Zaragoza, 204–205.

[B39] Martínez-Sánchez A Rojo S Rognes K Marcos-Garcia MA (1998) Califóridos con interés faunistico en agroecosistemas de dehesa y catálogo de las especies ibéricas de Polleniinae (Diptera: Calliphoridae). Boletín de la Asociación Española de Entomologia 22: 171–183.

[B40] Médail F Quézel P (1999) Biodiversity hotspots in the Mediterranean Basin: setting global conservation priorities. Conservation Biology 13: 1510–1513. doi: 10.1046/j.1523-1739.1999.98467.x

[B41] Michalski M Szpila K (in press) New data about distribution of *Chrysomya albiceps* (Diptera: Calliphoridae) in Poland. Dipteron.

[B42] Myers N Mittermeier R Mittermeier C Fonseca G Kent J (2000) Biodiversity hotspots for conservation priorities. Nature 403: 853–858. doi: 10.1038/35002501 10706275 10.1038/35002501

[B43] Neves H Valente A Faria B Silva I Marques J (1996) Laurissilva da Madeira – caracterização quantitativa e qualitativa. Parque Natural da Madeira, Grafimadeira, Funchal, 192 pp.

[B44] New TR (Ed.) (2012) Insect conservation: past, present and prospects. Springer, Dordrecht, 435 pp.

[B45] Norris KR (1965) The bionomics of blow flies. Annual Review of Entomology 10: 47–68. doi: 10.1146/annurev.en.10.010165.000403

[B46] Peck SB Heraty J Landry B Sinclair BJ (1998) The introduced insect fauna of an oceanic archipelago: the Galapagos Islands, Ecuador. American Entomologist 44: 218–237. doi: 10.1093/ae/44.4.218

[B47] Pérez-Bañón C Petanidou T Marcos-Garcia MA (2007) Pollination in small islands by occasional visitors: the case of Daucus carota subsp. commutatus (Apiaceae) in the Columbretes archipelago, Spain. Plant Ecology 192: 133–151. doi: 10.1007/s11258-006-9233-1

[B48] Peris SV (1952) La subfamilia Rhiniinae. Anales de la Estación Experimental de Aula Dei 3: 1–224.

[B49] Peris SV González-Mora D (1991) Los Calliphoridae de España, III: Luciliini (Diptera). Boletín Real Sociedad Española de História Natural (Sección Biológica) 87: 187–207.

[B50] Pombo AD Aguiar AMF Nunes E (2010) Exotic arthropods in Macaronesia: vectors, pathways, control measures and global trade. In: Serrano ARM Borges PAV Boieiro M Oromí P (Eds) Terrestrial arthropods of Macaronesia – biodiversity, ecology and evolution. Sociedade Portuguesa de Entomologia, Lisboa, 145–168.

[B51] Prado e Castro C Arnaldos MI García MD (2010) Additions to the Calliphoridae (Diptera) fauna from Portugal, with description of new records. Boletín de la Asociación Española de Entomología 33: 425–437.

[B52] Prado e Castro C García MD (2009) First record of *Chrysomya megacephala* (Fabricius, 1794) (Diptera, Calliphoridae) from Portugal. Graellsia 65: 75–77. doi: 10.3989/graellsia.2009.v65.i1.139

[B53] Prado e Castro C Serrano A Martins da Silva P García MD (2012) Carrion flies of forensic interest: a study of seasonal community composition and succession in Lisbon, Portugal. Medical and Veterinary Entomology 26: 417–431. doi: 10.1111/j.1365-2915.2012.01031.x 22765479 10.1111/j.1365-2915.2012.01031.x

[B54] Prado e Castro C Szpila K Rego C Boieiro M Serrano ARM (2016) First finding of larviposition in *Calliphora loewi* from an island relict forest. Entomological Science 19: 77–81. doi: 10.1111/ens.12163

[B55] Rognes K (1987) The taxonomy of the *Pollenia rudis* species-group in the Holarctic Region (Diptera: Calliphoridae). Systematic Entomology 12: 475–502. doi: 10.1111/j.1365-3113.1987.tb00219.x

[B56] Rognes K (1991) Blowflies (Diptera, Calliphoridae) of Fennoscandia and Denmark. Fauna Entomologica Scandinavica 24: 1–272.

[B57] Rognes K (1992) Revision of the cluster-flies of the *Pollenia venturii* species-group, with a cladistic analysis of Palaearctic species of *Pollenia* Robineau-Desvoidy (Diptera: Calliphoridae). Entomologica Scandinavica 23: 233–248. doi: 10.1163/187631292X00083

[B58] Rognes K (1998) Family Calliphoridae. In: Papp L Darvas B (Eds) Contributions to a Manual of Palaearctic Diptera: Higher Brachycera 3 Science Herald, Budapest, 617–648.

[B59] Rognes K (2013) Fauna Europaea: Calliphoridae. In: Pape T, Beuk P (Eds) Diptera: Brachycera. Fauna Europaea version 2.6.2, http://www.faunaeur.org [accessed 20–10–2016]

[B60] Rognes K Baz A (2007) A new species in the *Pollenia viatica* species-group from Sierra de Guadarrama, Spain (Diptera: Calliphoridae). Studia Dipterologica 14: 389–395.

[B61] Schumann H (1986) Family Calliphoridae. In: Soós Á Papp L (Eds) Catalogue of Palaearctic Diptera. Vol. 12. Calliphoridae – Sarcophagidae. Akadémiai Kiadó, Budapest, 11–59.

[B62] Serrano ARM Aguiar CAS Boieiro M Borges PAV Cardoso P Crespo L Farinha A Franquinho Aguiar AM Hortal J Martins da Silva P Menezes D Palma C Prado e Castro C Pereira F Rego C Ribeiro Silva P Santos AM Sousa JP (2014) Conflito entre actividades humanas e a conservação de endemismos insulares numa área de elevada biodiversidade à escala Mundial (ref. PTDC/BIA-BEC/99138/2008). Relatório final, Centro de Biologia Ambiental, Lisboa, Portugal, 67 pp.

[B63] Silva L Land EO Rodríguez Luengo JL (Eds) (2008) Invasive terrestrial flora and fauna of Macaronesia. Top 100 in Azores, Madeira and Canaries. Arena, Ponta Delgada, 545 pp http://cita.angra.uac.pt/ficheiros/publicacoes/1258549928.pdf

[B64] Singh B Wells JD (2013) Molecular systematics of the Calliphoridae (Diptera: Oestroidea): evidence from one mitochondrial and three nuclear genes. Journal of Medical Entomology 50(1): 15–23. doi: 10.1603/ME11288 23427647 10.1603/me11288

[B65] Smith KGV (1986) A manual of forensic entomology. The Trustees of the British Museum (Natural History), London, 205 pp.

[B66] Szpila K (2003) First instar larvae of nine West-Palaearctic species of *Pollenia* Robineau-Desvoidy, 1830 (Diptera, Calliphoridae). Entomologica Fennica 14: 193–210.

[B67] Szpila K (2012) Key for identification of European and Mediterranean blowflies (Diptera, Calliphoridae) of medical and veterinary importance – adult flies. In: Gennard D (Ed.) Forensic entomology, an introduction, II edition Willey-Blackwell, Chichester, 77–81.

[B68] Szpila K Hall MJR Pape T Grzywacz A (2013) Morphology and identification of first instars of the European and Mediterranean blowflies of forensic importance. Part II. Luciliinae. Medical and Veterinary Entomology 27: 349–366. doi: 10.1111/j.1365-2915.2012.01059.x 23205742 10.1111/j.1365-2915.2012.01059.x

[B69] Szpila K Matuszewski S Bajerlein D Konwerski S (2008) *Chrysomya albiceps* (Wiedemann, 1819), a forensically important blowfly (Diptera: Calliphoridae) new for the Polish fauna. Polish Journal of Entomology 77: 351–355.

[B70] Szpila K Pape T Hall MJR Mądra A (2014) Morphology and identification of first instars of European and Mediterranean blowflies of forensic importance. Part III: Calliphorinae. Medical and Veterinary Entomology 28: 133–142. doi: 10.1111/mve.12021 23834428 10.1111/mve.12021

[B71] Tantawi TI Whitworth TL Sinclair BJ (in press) Revision of the Nearctic *Calliphora* Robineau-Desvoidy (Diptera: Calliphoridae). Zootaxa. 10.11646/zootaxa.4226.3.1 28187619

[B72] Van Swaay C Cuttelod A Collins S Maes D Lopez Munguira M Šašić M Settele J Verovnik R Verstrael T Warren M Wiemers M Wynhof I (2010) European red list of butterflies. Publications Office of the European Union, Luxembourg, 47 pp http://ec.europa.eu/environment/nature/conservation/species/redlist/downloads/European_butterflies.pdf

[B73] Verdú JR Galante E (Eds) (2009) Atlas de los invertebrados amenazados de España (especies en peligro crítico y en peligro). Dirección General para la Biodiversidad, Ministerio de Medio Ambiente, Madrid, 340 pp http://www.magrama.gob.es/es/biodiversidad/temas/inventariosnacionales/atlas_invert_amenazados_espana_tcm7–21904.pdf

[B74] Verves YuG (2004) Records of *Chrysomya albiceps* in the Ukraine. Medical and Veterinary Entomology 18: 308–310. 15347401 10.1111/j.0269-283X.2004.00512.x

[B75] Verves YuG (2005) A catalogue of Oriental Calliphoridae (Diptera). Dipterological Research 16: 223–310. doi: 10.1111/j.0269-283X.2004.00512.x

[B76] Wall R Howard JJ Bindu J (2001) The seasonal abundance of blowflies infesting drying fish in south-west India. Journal of Applied Ecology 24: 223–227. doi: 10.1046/j.1365-2664.2001.00588.x

[B77] Wells J (1991) *Chrysomya megacephala* (Diptera: Calliphoridae) has reached the continental United States: review of its biology, pest status, and spread around the world. Journal of Medical Entomology 28: 471–473. doi: 10.1093/jmedent/28.3.471 1875378 10.1093/jmedent/28.3.471

[B78] Yahnke W George JA (1972) Rearing and immature stages of the cluster fly (*Pollenia rudis*) (Diptera: Calliphoridae) in Ontario. Canadian Entomologist 104: 567–576. doi: 10.4039/Ent104567-4

[B79] Zhang D Yan L Zhang M Chu H Cao J Li K Hu D Pape T (2016) Phylogenetic inference of calyptrates, with the first mitogenomes for Gasterophilinae (Diptera: Oestridae) and Paramacronychiinae (Diptera: Sarcophagidae). International Journal of Biological Science 12: 489–504. doi: 10.7150/ijbs.12148 10.7150/ijbs.12148 PMC4807417 27019632

[B80] Zumpt F (1965) Myiasis in man and animals in the Old World. Butterworths, London, 267 pp.

